# Functional Annotation and Identification of Candidate Disease Genes by Computational Analysis of Normal Tissue Gene Expression Data

**DOI:** 10.1371/journal.pone.0002439

**Published:** 2008-06-18

**Authors:** Laura Miozzi, Rosario Michael Piro, Fabio Rosa, Ugo Ala, Lorenzo Silengo, Ferdinando Di Cunto, Paolo Provero

**Affiliations:** 1 Institute of Plant Virology, CNR, Turin, Italy; 2 Molecular Biotechnology Center and Department of Genetics, Biology and Biochemistry, University of Turin, Torino, Italy; 3 ISI Foundation, Turin, Italy; Harvard School of Public Health, United States of America

## Abstract

**Background:**

High-throughput gene expression data can predict gene function through the “guilt by association” principle: coexpressed genes are likely to be functionally associated.

**Methodology/Principal Findings:**

We analyzed publicly available expression data on normal human tissues. The analysis is based on the integration of data obtained with two experimental platforms (microarrays and SAGE) and of various measures of dissimilarity between expression profiles. The building blocks of the procedure are the Ranked Coexpression Groups (RCG), small sets of tightly coexpressed genes which are analyzed in terms of functional annotation. Functionally characterized RCGs are selected by means of the majority rule and used to predict new functional annotations. Functionally characterized RCGs are enriched in groups of genes associated to similar phenotypes. We exploit this fact to find new candidate disease genes for many OMIM phenotypes of unknown molecular origin.

**Conclusions/Significance:**

We predict new functional annotations for many human genes, showing that the integration of different data sets and coexpression measures significantly improves the scope of the results. Combining gene expression data, functional annotation and known phenotype-gene associations we provide candidate genes for several genetic diseases of unknown molecular basis.

## Introduction

Among the open problems of molecular biology the functional annotation of the human genome and the identification of genes involved in genetic diseases are especially important. Expression data on a genomic scale have been available for several years thanks to various experimental techniques, and are widely believed to contain a wealth of information relevant to the solution of such problems.

Functional annotation based on expression data is usually founded on the “guilt by association” principle: since there is a strong correlation between coexpression and functional relatedness, a gene found to be coexpressed with several others involved in a given biological process can be predicted to be involved in the same process [1]–[3]. Recent systematic studies have demonstrated the soundness of the approach [4], [5].

Typically the analysis proceeds in three steps: (1) definition of a quantitative measure of dissimilarity between expression profiles, (2) identification of groups of coexpressed genes, *e.g.* using clustering algorithms (3) functional analysis of these groups using a controlled annotation vocabulary such as the Gene Ontology (GO) [6], [7].

In this work we analyze human normal tissues expression data with a procedure combining data obtained with different experimental techniques, and interpreted with different definitions of coexpression, into a unified framework. Thanks to a stringent definition of functional characterization this approach allows the generation of a large set of high-confidence predictions in terms of functional annotation and the identification of new candidate disease genes.

The distinctive features of our approach are:

- Integration of different datasets and measures of coexpression. The working hypothesis behind this strategy is that different experimental techniques and different definitions of dissimilarity measures explore different aspects of coexpression, and therefore can be combined to maximize the useful information obtained.
- Use of a rank-based procedure to generate groups of coexpressed genes (Ranked Coexpression Groups - RCG), without clustering algorithms.
- Use of the majority rule to determine the functional characterization of the RCGs. Such highly stringent criterion allows the generation of high-confidence functional predictions on the genes included in the functionally characterized RCGs.

The Ranked Coexpression Groups were generated from publicly available expression data on human normal tissues obtained with Affymetrix microarrays and SAGE; for the microarray data we used Euclidean distance and Pearson linear dissimilarity, while for SAGE we also used two measures of coexpression based on the Poisson distribution and originally introduced in [8] in a different context.

The RCGs determined to be functionally characterized by the majority rule were then used for two purposes:

1. to generate high-confidence functional predictions for the genes included in the functionally characterized RCGs
2. to identify promising new candidate disease genes for OMIM [9] phenotypes of unknown molecular basis, but for which one or more genetic loci have been identified. These predictions are based on the co-occurrence in functionally characterized RCGs of genes known to cause similar phenotypes

## Results and Discussion

### Ranked Coexpression Groups

In this work we considered gene expression data derived from human normal tissues with Affymetrix microarrays and with SAGE, but the techniques we employed are readily generalized to any high-throughput gene expression platform. Given a set of expression data and a quantitative measure of coexpression, for each gene in the dataset we defined a Ranked Coexpression Group as the gene itself together with the *k* genes most closely coexpressed with it.

Therefore from a gene expression dataset and a quantitative measure of coexpression we generated a number of RCGs equal to the number of genes in the dataset, each containing *k*+1 genes. Contrary to the clusters generated by most clustering algorithms, the RCGs can overlap each other.

### Generating putative functional annotations

We defined a RCG to be functionally characterized if the *majority* of the genes it contains share a functional annotation (GO term). If a RCG was found to be functionally characterized by a GO term, we assigned the same term to all the genes in the RCG which were not annotated to it: in this way we produced a set of predicted functional annotations.

The parameter *k* controls the sensitivity and specificity of the method: with increasing *k* one obtains less predictions with a smaller percentage of false positives (PFP, defined in the Methods) in the functional predictions. We chose the smallest value of *k* corresponding to an overall PFP of less than 1%, which turned out to be *k* = 6.

Our definition of functional characterization differs from the one most commonly used, in which a cluster of genes is considered functionally characterized if a GO term is significantly overrepresented among its member genes. Using the purely statistical definition, a GO term can turn out to be significantly overrepresented even if a relatively small fraction of the genes are annotated to it. In this case annotating the other genes to the term would probably generate a large fraction of false positives. Our majority rule ensures a higher level of confidence for the predicted annotations.

By limiting ourselves to Gene Ontology terms whose total prevalence in all the genes included in the expression dataset is lower than a given threshold *M*, we can make the majority rules imply statistical overrepresentation. For example the probesets of the Affymetrix data set we use can be associated to 18099 unique genes: since we only consider GO terms whose prevalence among these genes is ≤*M* = 300, the situation of least statistical significance is that of a RCG with 4 out of 7 genes annotated to a term with total prevalence of 300, which corresponds to a P-value of 2.5 10^−6^ (one-sided exact Fisher test). The converse is not true, since for example a GO term whose total prevalence is 2, found twice in a RCG, would be statistically overrepresented with P-value 1.35 10^−7^ but would not be selected by the majority rule.

### Datasets and measures of coexpression

We used human normal tissue gene expression data obtained with Affymetrix and SAGE platforms. The Affymetrix data were produced by the authors of Ref.[10] and deposited in the GEO repository [11], [12] under accession GSE3526 [13]. 353 microarray experiments were performed on ten post-mortem donors, representing 65 tissues including 20 distinct sites of the central nervous system. On these data we used Euclidean distance and Pearson linear dissimilarity as measures of coexpression.

The SAGE data were obtained from the CGAP web site [14], limiting the download to the 66 libraries labeled as “normal tissue”. On the SAGE data we used Pearson linear dissimilarity, Euclidean distance, and two different Poisson-based coexpression measures introduced in Ref.[8], originally to define a similarity measure between promoters based on counts of transcription factor binding sites.

Each dataset/coexpression measure gave its own set of RCGs and the corresponding predicted annotations. Table 1 shows the number of predicted annotations obtained with the various combinations, together with an estimate of the PFP obtained as explained in the Methods section.

**Table 1:** Predicted annotations.

dataset	measure	annotations	PFP(%)
AFFY	P	1882	0.26
AFFY	E	925	0.61
SAGE	P	738	1.26
SAGE	E	374	2.53
SAGE	D	414	2.33
SAGE	O	539	1.70

Number of non-redundant predicted annotations obtained with the various dataset/measure combinations, and the corresponding PFP. The coexpression measures are: **E**: Euclidean; **P**: Pearson; **D**: Poisson “distinct”; **O**: Poisson “overrepresentation”.

It would be possible to conclude from these data that, for example, Pearson dissimilarity is the best predictor of functional relatedness both for Affymetrix and SAGE data. However it should be noted that:

- The signal to noise ratio is rather small for each dataset/measure combination separately, as shown by the PFP values shown in Table 1; and
- The overlap between the predicted annotations obtained with different combinations is rather limited (Table 2). For example the predictions obtained from microarray data and Euclidean distance are definitely not a subset of the more numerous ones obtained with Pearson dissimilarity.

**Table 2:** Overlap.

	AFFY P	AFFY E	SAGE P	SAGE E	SAGE D	SAGE O
AFFY P	*1882*	168	38	20	11	21
AFFY E		*925*	16	13	6	15
SAGE P			*738*	58	46	65
SAGE E				*374*	45	69
SAGE D					*414*	201
SAGE O						*539*

Overlap between the predicted annotations found by the various dataset/measure combinations. E: Euclidean, P: Pearson, D: Poisson-distinct, O: Poisson-overrepresentation.

These facts indicate that different combinations are able to usefully explore different aspects of coexpression: therefore the most effective strategy is to consider the *union* of all such predictions.

In particular, the small overlap between the predictions obtained with Affymetrix and SAGE data is in agreement with the results of Ref. [15]. In that paper it is shown, more generally, that the overlap between pairs of coexpressed genes from Affymetrix and SAGE experiments is significant but small. Our results show however that data from both platform can be used to obtain good quality functional prediction if a very stringent selection is performed, such as the one defined by the majority rule applied to RCGs.

Ref. [15] also provides a study of the “GO correlation” (fraction of pairs of coexpressed genes sharing their most specific GO annotation) as a function of the cutoff in Pearson correlation coefficient used to define coexpression. For example, they observe a very strong enrichment in GO correlation when the cutoff is set at values of 0.8 or higher. For comparison, the average Pearson correlation in all our Affymetrix (SAGE) RCGs is 0.785 (0.711), while for functionally characterized ones it is 0.868 (0.836).

The result of our analysis is thus the union of the predicted annotations produced by each combination, a total of 4058 non-redundant associations between a gene and a GO term involving 2440 human genes and 255 GO terms (non-redundant means that when the same gene was annotated to a term and to a descendant of the same term in the GO graph, only the latter annotation was retained). The PFP for this set of predictions can be estimated as the weighted average of the PFPs of the single combinations to be 0.987%. The functional predictions are reported in Supporting Information Text S1.

### High-confidence annotations

While the PFP gives a general statistical measure of the reliability of our predictions, it is possible to extract a high-confidence subset of predicted annotations by estimating the precision associated to each individual GO term through a leave-one-out procedure.

First we produced, for each GO term, a list of *recalled* annotations, that is a list of genes that are known to be associated to the GO term and would be associated to the same term by the method if the known association were to be ignored. Then, as in [4], we defined the precision as the ratio between the number of recalled associations and the total number of recalled and predicted associations between annotated genes and the GO term.

We generated a list of GO terms with precision ≥50% for each dataset/measure combination. Putting together the corresponding functional predictions and eliminating redundancies, we ended up with 758 high-confidence annotations involving 510 genes and 50 GO terms (Supporting Information Text S2)

### Comparison with a SVM-based approach

In Ref. [4] a method based on a Support Vector Machine (SVM) was introduced to solve the same problem, *i.e.* to generate genome-wide predicted functional annotations based on normal-tissue expression data. The authors produced a list of 3730 putative annotations with a precision of 50% or better. After removing redundancies and selecting, as in our case, only the GO terms with total prevalence of 300 or less, we are left with 1750 putative annotations, to be compared to the 758 high-confidence annotations we obtained.

Such a difference could in principle be due to higher effectiveness of the SVM method compared to the majority rule, or to differences in the primary data used in the analysis, or both. Therefore we decided to use our algorithm on the data used in [4], with the known GO annotations provided in the supplementary material of the same work. We proceeded exactly as described before, using Pearson dissimilarity and Euclidean distance, and we obtained a total of 1990 high-confidence annotations (Supporting Information Text S3). Therefore, at least on this expression dataset, the performance of the majority rule method is similar to the SVM. Interestingly, 292 of our 1990 predictions are in common with the SVM predictions. Such moderate degree of overlap suggests that the two methods can explore different aspects of the same expression data, and can therefore be considered as complementary.

However our method has two main advantages over the use of SVMs or other complex machine learning algorithms: first, it is simple to implement and does not require much computational power; and second, the RCGs, and in particular the functionally characterized ones, can be further mined to obtain insight on other biological problems. For example, in the next section we show how RCGs can be used to predict new candidate disease genes.

### Using RCGs to find candidate disease genes

Since the RCGs could successfully be used to predict functional annotations we asked whether they could also be useful in finding new candidate disease genes. First, we verified that genes whose mutations are known to cause similar phenotypes tend to appear together in RCGs more often than expected by chance. We used the similarity score between OMIM phenotypes developed in [16], and we defined as similar two phenotypes with a similarity score ≥0.4, as suggested in [16].

**Figure 1:**
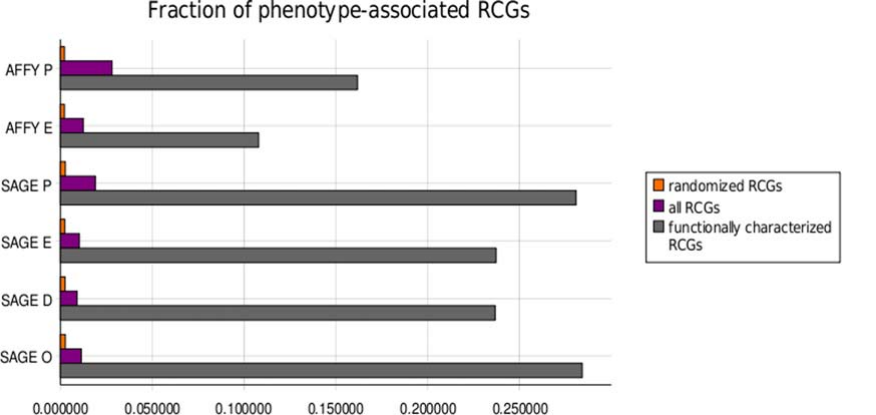
Enrichment of RCGs in genes involved in similar phenotypes. For each dataset/coexpresison measure combination we show the fraction of RCGs associated to an OMIM phenotype as described in the text. The fraction is shown for functionally characterized RCGs (grey), all RCGs (purple), and randomized RCGs (orange). The coexpression measures are: E: Euclidean; P: Pearson; D: Poisson “distinct”; O: Poisson “overrepresentation”.

For each dataset/measure combination we counted the fraction of RCGs containing at least two genes associated to similar phenotypes, and compared it to the same fraction obtained after randomization of the gene names. The results are shown in Fig. 1. All dataset/measure combinations showed a significant enrichment of disease-associated RCGs compared to randomized RCGs. Moreover, this enrichment was much larger for functionally characterized RCGs compared to all RCGs (as expected, since functional annotation and involvment in genetic diseases are correlated).

These results suggest that RCGs, and especially functionally characterized ones, can be used to predict new candidate genes for those phenotypes for which only a genomic locus, and not an individual gene, is known. We considered a gene to be a candidate for a phenotype if

- its genomic location fell within a locus known to be associated to the phenotype; and
- the gene belonged to a functionally characterized RCG containing at least two genes known to cause similar phenotypes.

We obtained a total of 53 candidate disease genes for 33 orphan loci (Table 3 and Supporting Information Text S4). A survey of the corresponding OMIM records and the literature revealed that 18 of our candidates could have been selected as very likely on the basis of the available evidence, because they are known to be involved in phenotypes very similar to those associated to the orphan loci. Seven of these cases have been already excluded, on a purely clinical basis, because a mutation was found in a different gene or because of a negative mutation screening. Nevertheless, since mutations are usually searched for only within the annotated exons, we believe that the decision to definitively rule out the involvement of such genes should be taken cautiously. In these cases, our results can be considered as further, independent evidence which may justify the search for mutations in the regulatory regions of the genes or the re-evaluation of possible synonymous changes which may have been discovered in patients, since they could in principle cause aberrant splicings [17], [18].

**Table 3:** Predicted disease genes.

Gene	Phenotype	Status
PCOLCE	Ehlers-Danlos syndrome, type VIII	A
KCNQ2	Electroencephalogram, low-voltage	B*[24]
CACNA1H	Microphthalmia, isolated, with cataract 1	A
MYF6	Scapuloperoneal myopathy	A
COL2A1	Spondylometaphyseal dysplasia, Kozlowski type	B#[25]
PSMB5	Basal ganglia calcification, idiopathic, 1	A
NEDD8	Basal ganglia calcification, idiopathic, 1	A
C14orf2	Basal ganglia calcification, idiopathic, 1	A
ENSG00000196992	Basal ganglia calcification, idiopathic, 1	A
RSHL3	Craniometaphyseal dysplasia, autosomal recessive	A
SC5DL	Rosselli-Gulienetti syndrome	A
DKFZp762E1312	Holoprosencephaly	A
TULP1	Spinocerebellar ataxia, autosomal recessive 3	A
NRSN1	Spinocerebellar ataxia, autosomal recessive 3	A
OPN1MW	Microphthalmia, isolated, with coloboma 1	B
OPN1MW2	Microphthalmia, isolated, with coloboma 1	B
CACNA1F	Albinism, ocular, type II	B#[26]
OPN1LW	Colorblindness, blue-mono-cone-monochromatic type	B
OPN1MW	Colorblindness, blue-mono-cone-monochromatic type	B
OPN1MW2	Colorblindness, blue-mono-cone-monochromatic type	B
NRK	Megalocornea	A
MYL2	Spinal muscular atrophy, distal, congenital nonprogressive	A
TDG	Spinal muscular atrophy, distal, congenital nonprogressive	A
CAMK1D	Refsum disease with increased pipecolic acidemia	A
AIPL1	Cone-rod dystrophy 5	C$[27]
LOC257039	Glaucoma 1, open angle	A
BFSP2	Glaucoma 1, open angle	A
SLC35A1	Retinitis pigmentosa 25	A
IMPG1	Cone-rod dystrophy 7	A
ELOVL4	Cone-rod dystrophy 7	B$[28]
CNGA1	Stargardt disease 4	C
SAA4	Hyperlipidemia, combined, 2	A
CYP4F22	Ichthyosis, nonlamellar and nonerythrodermic, congenital, autosomal	A
ACTA1	Muscular dystrophy, congenital, 1b	B
CABC1	Muscular dystrophy, congenital, 1b	A
APOA5	High density lipoprotein deficiency, 3	B
APOA4	High density lipoprotein deficiency, 3	B
BFSP1	Cataract, posterior polar, 3	B
PCP4L1	Cone-rod dystrophy, 8	A
ABCF3	Abdominal obesity-metabolic syndrome	A
FOXE1	Cataract, autosomal recessive, early-onset, pulverulent	A
MYOT	Myopathy, distal 2	B%[29]
LOC493869	Myopathy, distal 2	A
CMYA5	Myopathy, distal 2	A
KRT1	Exfoliative ichthyosis, autosomal recessive	B
KRT75	Exfoliative ichthyosis, autosomal recessive	A
KRT2	Exfoliative ichthyosis, autosomal recessive	B
KRT5	Exfoliative ichthyosis, autosomal recessive	B
KRT77	Exfoliative ichthyosis, autosomal recessive	A
KRT6A	Exfoliative ichthyosis, autosomal recessive	A
FLNC	Muscular dystrophy, limb-girdle, type 1f	A
CRYBB1	Myopia 6	A
CRYBA4	Myopia 6	A

The gene name is the official HUGO name where available, or the Ensembl ID. The third column summarizes the available information about the association of candidates with the disease. In particular, genes annotated with **A** have to our knowledge not been associated to the disease or to similar phenotypes, genes annotated with **B** are involved in MIM phenotypes with phenomap scores of 0.4 or higher, genes annotated with **C** have been associated to similar phenotypes, but display a phenomap score lower than 0.4. Moreover, genes annotated with **#** represent the actual disease gene, because mutations have been found in patients (but the association with the disease is not reported by Ensembl, version 45); genes annotated with ^*^ have been excluded on clinical basis; genes annotated with **$** can be excluded because mutations have been found in a different gene; genes annotated **%** are at the moment excluded because they have been screened but mutations have not been found. In all cases labeled by special characters we also provide a reference to the corresponding supporting evidence. More details are available in Supporting Information Text S4.

In all of the other cases, our results represent completely new and extremely focused working hypotheses about the possible genetic origin and molecular basis of the mapped phenotype, of which we report some remarkable examples.

Rosselli-Gulienetti (RG) syndrome (OMIM ID 225000) is a very rare form of ectodermal dysplasia, associated with craniofacial abnormalities such as cleft palate and lip, whose molecular basis is completely unknown. Owing to phenotypic similarity with Desmosterolosis (602398) and Smith-Lemli-Opitz Syndrome (270400), our analysis provided Lathosterol 5-desaturase (SC5DL) as a candidate for this disorder. Although missense mutations in SC5DL are known to result in lathosterolosis, the complete inactivation of the gene in mice led to a malformative syndrome characterized by craniofacial defects, including cleft palate and micrognathia, and limb patterning abnormalities [19]. These malformations were consistent with impaired hedgehog signaling and appeared to be a consequence of decreased cholesterol rather than increased lathosterol [19]. Taken together, these facts strongly suggest that null SC5DL mutations could cause RG syndrome in humans.

Idiopathic basal ganglia calcification (IBGC1 - OMIM ID 213600) is a disorder of unknown origin mapped to Chr. 14q, a locus that comprises 1250 potential candidates. Our analysis suggests that this disorder is caused by mitochondrial disfunction, and strongly restricts the field of likely candidate genes.

The candidate that we provide for autosomal nonlamellar and nonerythrodermic ichthyosis (OMIM ID 604781) is CYP4F22, a cytochrome P450 family member of unknown function that we functionally associated with epidermal development. This result is particularly interesting in the light of recent results showing that another member of the family is implicated in a different form of ichthyosis [20].

Finally, one of the predicted disease/gene associations strongly suggests that our method could be of help in the identification of genes involved in highly prevalent clinical phenotypes. Indeed, a Quantitative Trait Locus for abdominal obesity (OMIM ID 605552) has been mapped on 3q27. The candidate that we provide for this locus is ABCF3, an ATP-binding cassette-containing gene of unknown function. Interestingly, on the basis of our predicted annotation, this gene could be involved in the synthesis of C21-steroid hormones, thus providing a strong rationale for its potential involvement and for searching variants of its sequence in obesity.

### Conclusions

We have investigated publicly available gene expression data to predict the function of human genes based on the guilt-by-association principle. A new method based on small groups of tightly coexpressed genes and the majority rule to obtain functional predictions was used on various combinations of datasets and measures of coexpression.

Even if it would be possible to rank these combinations by their effectiveness in producing putative annotations, we showed that the most effective approach is to integrate their results, since each dataset/measure combination provides a high signal to noise ratio, while the overlap between the results of the different combinations is limited.

While the method is simple and its implementation does not require large computational power, we showed that it performs similarly to a published method based on support vector machines. The Ranked Coexpression Groups on which the method is based can be used to gain insight into biological problems other than functional annotation: as an example we used them to identify new candidate disease genes.

Our main results are

- a set of 4058 non–redundant functional predictions, including a subset of 758 high-confidence predictions; and
- a set of 53 candidate disease genes associated to 33 OMIM loci with unknown molecular basis.

These results emphasize the crucial role that high-throughput gene expression data can play in solving the outstanding problems of molecular biology.

## Methods

### Expression data: microarray

Human normal tissue gene expression data generated by the authors of Ref.[10] were downloaded from the Gene Expression Omnibus [11], [12]. The data were log-transformed and replicate experiments corresponding to the same tissue were averaged, to compensate for the highly variable number of replicate experiments available for the 65 tissues considered.

The association between probesets and genes was obtained from Ensembl [21], [22], version 45. Logarithmic expression data for probesets corresponding to the same Ensembl gene id were averaged, while probesets not associated to any Ensembl id were discarded. We thus obtained an expression matrix with 18099 rows (Ensembl gene ids) and 65 columns (tissues), which was used to construct the RCGs.

### Expression data: SAGE

We used the SAGE library finder [23] to select all short-tag libraries with normal tissue histology, obtaining 66 libraries, and we downloaded the corresponding tag frequencies. An integer-valued expression matrix was obtained by summing the tag counts for all tags corresponding to the same Ensembl id. The translation from tag to Ensembl id was performed using the tag-Unigene id correspondence provided in the SAGE ftp site. We thus obtained an expression matrix with 14031 rows and 66 columns, which was used to construct the RCGs.

### GO annotation

GO annotations for Ensembl gene ids were obtained from Ensembl, version 45. Annotations with the “IEP” evidence code were discarded as they refer to annotations inferred from expression profiles, that is based on approaches similar to the one used in this work.

### Measures of coexpression

Given two genes in one of our expression matrices we used different quantitative measures of coexpression to construct independent sets of RCGs. Let *X* and *Y* be two genes, and their expression values for the *N* columns of the matrix (tissues). The expression data are real numbers for microarray data and integer counts for SAGE.

The Pearson linear dissimilarity is defined as
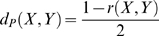
where *r* is the Pearson correlation coefficient. This measure of coexpression was used for both microarray and SAGE data.

The Euclidean distance is
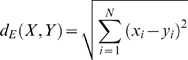
Also this measure was applied to both microarray and SAGE data. However the SAGE data were first normalized by dividing each tag count by the total number of tags counted in the library.

We also used, on SAGE data, two measures of coexpression based on the null hypothesis of Poissonian distribution of the tag counts. These were proposed in Ref.[8] in a different context, namely as a measure of similarity between promoter regions based on transcription factor binding site counts. In particular we used the two “dissimilarity metrics” introduced in Ref. [8].

They are both based on the null model in which the gene expression levels for the *i*-th SAGE library follow a Poisson distribution with expectation value given by the average gene expression in the library.

The “dissimilarity-distinct” measure is defined by
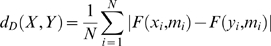
where *F*(*x*,*m*) is the Poisson distribution function, *i.e.* the probability of observing ≤*x* occurrences if the expected number of occurrences is *m*:
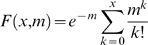
Therefore *d_D_*(*X*,*Y*) gets from each library a contribution equal to the area under the Poisson curve between *x_i_* and *y_i_*.

The “dissimilarity-overrepresentation” measure is based on the idea of overrepresentation of a gene in a library. If the expression level of gene *X* in the *i*-th library is *x_i_*, its overrepresentation is measured by the Poissonian probability of having at least *x_i_* occurrences:
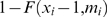

*d_O_*(*X*, *Y*) gets from each library a contribution equal to the difference in overrepresentation between *X* and *Y* in the library:
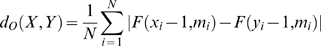
While the equations defining *d_D_* and *d_O_* look rather similar, it turns out *a posteriori* that the RCGs and putative annotations they produce are significantly different (see Table 2).

Other Poisson-based similarity measures are defined in [8], which however are not suitable for the generation of RCGs since, in practice, large number of genes turn out to have the same coexpression with any given gene, so as to make the definition of small RCGs problematic.

### Estimating the percentage of false positives and choosing *k*

For each combination of expression data and coexpression measure we estimated the percentage of false positives (PFP) among the putative gene annotations using randomized RCGs: we randomized the gene names 100 times independently and recorded the number of putative annotations obtained from each set of randomized RCGs. The PFP for a given data/measure combination is the ratio between the average number of predicted annotations obtained from the randomized RCGs and the number obtained from the true RCGs. The overall PFP is computed as the average over the PFPs of the various combinations, each weighted by the corresponding number of predicted annotations. When increasing the value of *k* one systematically obtains less predictions and a lower PFP. We decided to use the smallest *k* giving an overall PFP less than 1%, which turned out to be *k* = 6. For comparison, the PFP for *k* = 4 was 8.0%.

### Identification of candidate disease genes

To verify the enrichment of RCGs in pairs of genes involved in similar phenotypes we downloaded from Ensembl a list of associations between human genes and phenotypes known to be caused by their mutations. We defined two OMIM phenotypes to be similar if their MimMiner [16] similarity score was ≥0.4. We then computed the number of functionally characterized RCGs including at least two genes associated to similar phenotypes and compared it to the same number (a) for all RCGs and (b) averaged over 100 randomized RCGs to produce Figure 1.

A list of 850 OMIM phenotypes with unknown molecular basis, but for which one or more genomic loci have been identified, was obtained from the OMIM site [9] on July 2nd, 2007. For the 602 such phenotypes for which MimMiner scores were available we identified the functionally characterized RCGs containing at least two genes experimentally associated to similar phenotypes. Genes in such RCGs, which were also located inside one of the genomic loci associated to the phenotype of unknown molecular basis were considered as candidate genes for the phenotype.

## Supporting Information

Text S1 Non-redundant functional predictions for human genes.
(0.80 MB TXT)

Text S2 High-confidence non-redundant functional predictions for human genes.
(0.14 MB TXT)

Text S3 High-confidence non-redundant functional predictions for mouse genes based on the data of Ref.[4]
(0.20 MB TXT)

Text S4 Candidate genes for OMIM phenotypes of unknown molecular basis. The first column reports the dataset/measure combination(s) that allowed the selection of the candidate: 1 - Pearson correlation coefficient on Affymetrix dataset; 2 - Euclidean distance on Affymetrix dataset; 3 - Pearson correlation coefficient on SAGE dataset; 4 - normalized Euclidean distance on SAGE; 5 - Poisson-based “distinct” dissimilarity on SAGE; 6 - Poisson-based “overrepresentation” dissimilarity on SAGE. Codes for the available knowledge as in Tab. 3. The last column reports the GO annotations associated to the functionally characterized RCG(s) used to make the prediction
(0.09 MB XLS)

## References

[1] Brown PO, Botstein D (1999). Exploring the new world of the genome with DNA microarrays.. Nat Genet.

[2] Quackenbush J (2001). Computational analysis of microarraydata.. Nat Rev Genet.

[3] Eisen MB, Spellman PT, Brown PO, Botstein D (1998). Cluster analysis and display of genome-wide expression patterns.. Proc Natl Acad Sci U S A.

[4] Zhang W, Morris QD, Chang R, Shai O, Bakowski MA (2004). The functional landscape of mouse gene expression.. J Biol.

[5] Wolfe CJ, Kohane IS, Butte AJ (2005). Systematic survey reveals general applicability of guilt-by-association within gene coexpression networks.. BMC Bioinformatics.

[6] Ashburner M, Ball CA, Blake JA, Botstein D, Butler H (2000). Gene ontology: tool for the unification of biology. The Gene Ontology Consortium.. Nat Genet.

[7] The Gene Ontology.. http://www.geneontology.org/.

[8] van Helden J (2004). Metrics for comparing regulatory sequences on the basis of pattern counts.. Bioinformatics.

[9] Online Mendelian Inheritance in Man.. http://www.ncbi.nlm.nih.gov/omim/.

[10] Roth RB, Hevezi P, Lee J, Willhite D, Lechner SM (2006). Gene expression analyses reveal molecular relationships among 20 regions of the human CNS.. Neurogenetics.

[11] Barrett T, Troup DB, Wilhite SE, Ledoux P, Rudnev D (2007). NCBI GEO: mining tens of millions of expression profiles–database and tools update.. Nucleic Acids Res.

[12] Edgar R, Domrachev M, Lash AE (2002). Gene Expression Omnibus: NCBI gene expression and hybridization array data repository.. Nucleic Acids Res.

[13] Comparison of gene expression profiles across the normal human body.. http://www.ncbi.nlm.nih.gov/geo/query/acc.cgiaccGSE3526.

[14] The Cancer Genome Anatomy Project.. http://cgap.nci.nih.gov/.

[15] Griffith OL, Pleasance ED, Fulton DL, Oveisi M, Ester M (2005). Assessment and integration of publicly available SAGE, cDNA microarray and oligonucleotide microarray expression data for global coexpression analyses.. Genomics.

[16] van Driel MA, Bruggeman J, Vriend G, Brunner HG, Leunissen JAM (2006). A text-mining analysis of the human phenome.. Eur J Hum Genet.

[17] Faustino NA, Cooper TA (2003). Pre-mRNA splicing and human disease.. Genes Dev.

[18] Pagani F, Raponi M, Baralle FE (2005). Synonymous mutations in CFTR exon 12 affect splicing and are not neutral in evolution.. Proc Natl Acad Sci U S A.

[19] Krakowiak PA, Wassif CA, Kratz L, Cozma D, Kovarova M (2003). Lathosterolosis: an inborn error of human and murine cholesterol synthesis due to lathosterol 5-desaturase deficiency.. Hum Mol Genet.

[20] Lefevre C, Bouadjar B, Ferrand V, Tadini G, Megarbane A (2006). Mutations in a new cytochrome P450 gene in lamellar ichthyosis type 3.. Hum Mol Genet.

[21] Hubbard TJP, Aken BL, Beal K, Ballester B, Caccamo M (2007). Ensembl 2007.. Nucleic Acids Res.

[22] Ensembl.. http://www.ensembl.org/.

[23] Sage Library Finder.. http://cgap.nci.nih.gov/SAGE/SAGELibraryFinder.

[24] Steinlein O, Anokhin A, Yping M, Schalt E, Vogel F (1992). Localization of a gene for the human low-voltage EEG on 20q and genetic heterogeneity.. Genomics.

[25] Sulko J, Czarny-Ratajczak M, Wozniak A, Latos-Bielenska A, Kozlowski K (2005). Novel amino acid substitution in the Y-position of collagen type II causes spondyloepimetaphyseal dysplasia congenita.. Am J Med Genet A.

[26] Wutz K, Sauer C, Zrenner E, Lorenz B, Alitalo T (2002). Thirty distinct CACNA1F mutations in 33 families with incomplete type of XLCSNB and Cacna1f expression profiling in mouse retina.. Eur J Hum Genet.

[27] Udar N, Yelchits S, Chalukya M, Yellore V, Nusinowitz S (2003). Identification of GUCY2D gene mutations in CORD5 families and evidence of incomplete penetrance.. Hum Mutat.

[28] Michaelides M, Holder GE, Hunt DM, Fitzke FW, Bird AC (2005). A detailed study of the phenotype of an autosomal dominant cone-rod dystrophy (CORD7) associated with mutation in the gene for RIM1.. Br J Ophthalmol.

[29] Garvey SM, Senderek J, Beckmann JS, Seboun E, Jackson CE (2006). Myotilin is not the causative gene for vocal cord and pharyngeal weakness with distal myopathy (VCPDM).. Ann Hum Genet.

